# Initial Normotensive Presentation of a Primigravida With Posterior Reversible Encephalopathy Syndrome: A Case Report

**DOI:** 10.7759/cureus.19407

**Published:** 2021-11-09

**Authors:** Angelica Aduen-Carrillo, Maria Jose Hernandez-Woodbine, Camilo A Avendaño-Capriles, Francisco N Ayola-Anaya

**Affiliations:** 1 Critical Care, Medicina Alta Complejidad, Barranquilla, COL; 2 Department of Medicine, Universidad del Norte, Barranquilla, COL

**Keywords:** brain diseases, case report, pregnancy, eclampsia, pre-eclampsia

## Abstract

Posterior reversible encephalopathy syndrome (PRES) is a clinical-radiological entity characterized by variable neurological manifestations, primarily caused by pathophysiological changes related to cerebral autoregulation that result in radiologically evident vasogenic edema. It is usually associated with hypertensive states, but it is not exclusively related to those.

A healthy 18-year-old primigravid woman with no proteinuria or hypertension on admission presented with normotensive PRES. She had an intense diffuse headache that preceded a generalized tonic-clonic seizure. Her neurological status deteriorated, and hypertension was detected afterward. Brain imaging revealed bilateral vasogenic edema in the occipital region. Magnesium sulfate and antihypertensive medications were administered. A cesarean section was performed, and her neurological symptoms subsequently improved, leading to discharge with no complications.

This case highlights the importance of suspecting PRES in pregnant patients even in the absence of preeclampsia.

## Introduction

The hypertensive disorders of pregnancy (HDP) - chronic hypertension, gestational hypertension, and preeclampsia/eclampsia - remain one of the major causes of maternal mortality and morbidity worldwide, including in Latin America and the Caribbean. HDP is associated with an increased risk of cardiovascular and renal diseases even long after delivery. A higher risk of HDP has been described in primiparous women compared to multiparous women [1].

Posterior reversible encephalopathy syndrome (PRES) is a clinical-radiological condition characterized by neurological manifestations associated with posterior cerebral edema, usually transient. Its incidence in the general population is unknown, and its signs and symptoms can vary according to the severity. However, some patients may present with headaches, visual disturbances, altered consciousness, and generalized tonic-clonic seizures [2,3].

PRES is usually associated with hypertension, preeclampsia, and eclampsia but can also occur in the setting of infection/sepsis/shock, autoimmune diseases, chemotherapy, and transplantation. It is more common in females and middle-aged adults, but it may be underdiagnosed in other population groups. Radiologically, bilateral symmetric vasogenic edema has been described in the territories of the posterior cerebral circulation [2,4].

In this report, we present a case of a young female patient diagnosed with PRES in the absence of preeclampsia, prompting clinicians to suspect it in atypical scenarios.

## Case presentation

A healthy 18-year-old G1P0, 28 weeks' gestational age by first-trimester ultrasound with no previous prenatal care, was admitted to the obstetric intensive care unit of a hospital in Barranquilla (Colombia). She was evaluated for a two-day history of intense diffuse headache and grade II lower limb edema, preceding a generalized tonic-clonic seizure that lasted approximately three minutes without any other associated sign or symptom. Physical examination performed on admission revealed eye-opening in response to verbal command, incomprehensible speech, withdrawal to pain, and mildly reactive isochoric pupils [Glasgow Coma Scale (GCS) score: 9/15]. Vital signs were within normal limits (blood pressure was 118/72 mmHg).

Eclampsia or previously uncontrolled epilepsy were among our differentials. At the time, magnesium sulfate was initiated for neuroprotection. Later, our patient’s neurological status deteriorated (GCS score: 8/15), requiring endotracheal intubation. Furthermore, she developed new-onset elevated blood pressure (168/112 mmHg), which required intravenous antihypertensive management (labetalol 20 mg intravenously over two minutes followed by infusion of 1 mg/minute). Toxicological panels, immunologic workup, lumbar puncture, hematologic profile, urinary protein determination, and obstetric Doppler ultrasound were requested, which yielded normal results. Measurement of angiogenic and antiangiogenic factors was performed with results above the normal limits (Table 1).

**Table 1:** Angiogenic and antiangiogenic factors

Angiogenic and antiangiogenic factors							
Soluble fms-like tyrosine kinase-1 (sFlt-1)			4389			pg/ml	
Theoretical values:							
Percentiles of the sFlt-1 test (pg/ml)							
Weeks of gestation							
	10 - 14	15 - 19	20 - 23	24 - 28	29 - 33	34 - 36	37 - delivery
5th percentile	555	470	649	630	707	978	1671
50th percentile	1445	1459	1576	1449	1934	2972	4400
95th percentile	2361	2785	2944	3890	6688	9921	11324
Placental growth factor (PIGF)			19.8			pg/ml	
Theoretical values:							
Percentiles of the PIGF test (pg/ml)							
Weeks of gestation							
	10 - 14	15 - 19	20 - 23	24 - 28	29 - 33	34 - 36	37 - delivery
5th percentile	29.4	65.7	125	130	73.3	62.7	52.3
50th percentile	62.8	135	265	412	439	232	161
95th percentile	183	203	541	1108	1108	972	659
Ratio between sFlt-1/PIGF percentiles			221.7				
Theoretical values:							
sFlt-1/PIGF ratio							
Weeks of gestation							
	10 - 14	15 - 19	20 - 23	24 - 28	29 - 33	34 - 36	37 - delivery
5th percentile	5.21	4.32	2.19	1.01	0.945	1.38	3.65
50th percentile	22.7	12.6	6.09	3.80	4.03	13.3	26.2
95th percentile	57.3	26.9	14.8	16.9	86.4	92	138
Percentile interpretation							
5th percentile	Low						
50th percentile	Normal						
95th percentile	High						

Brain CT scan showed a poorly defined hypodense area and bilateral vasogenic edema in the occipital region, suggesting PRES. After 48 hours, her neurological status improved, although elevated blood pressure persisted. No other convulsive crisis or vasomotor symptoms were observed. A cesarean section was performed with no complications.

**Figure 1:**
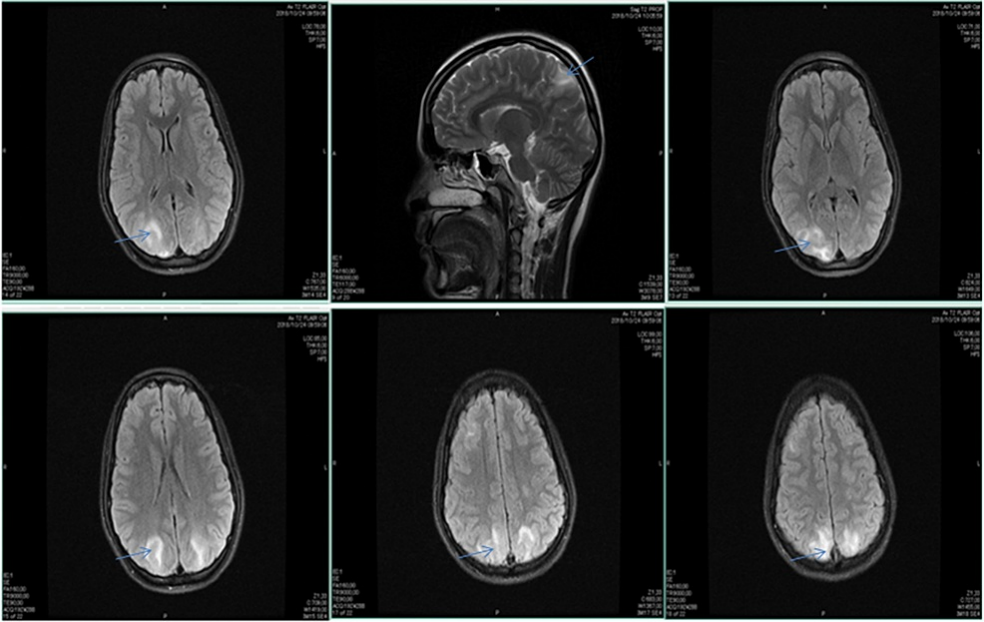
Cerebral MRI T2-weighted spin-echo and gradient MRI sequences in axial and sagittal planes, with subcortical T2 signal enhancement in the posterior temporal and occipital regions indicated with straight arrows. This signal increase is compatible with edema, predominantly vasogenic. In T2 gradient sequences, there was no evidence of hemorrhagic lesions MRI: magnetic resonance imaging

Cerebral MRI showed subcortical white matter edema in the posterior cerebral area, primarily on parieto-occipital regions and the right hemisphere. In addition to the clinical history and examination, these findings confirmed the PRES diagnosis in the patient (Figure 1). After a week, blood pressure levels were controlled with antihypertensive medications, and the neurological status remained favorable. Subsequently, the patient was discharged from our institution with recommendations and a follow-up appointment.

## Discussion

We discussed a previously healthy primigravid female with an initially normotensive presentation of a generalized tonic-clonic seizure. Eclampsia can be defined as the convulsive manifestation of preeclampsia. Preeclampsia refers to the new onset of hypertension accompanied by either proteinuria or end-organ dysfunction after 20 weeks of gestation in a previously normotensive woman [5]. In this patient, typical clinical syndrome and neuroimaging findings of PRES indicated eclampsia, even though proteinuria and hypertension were absent on admission.

Women at higher risk of this condition are usually non-white, nulliparous, and from lower socioeconomic backgrounds, all of which were characteristics of our patient. PRES has been described in the context of multiple medical scenarios like hypertensive encephalopathy and eclampsia. Since it is typically reversible, early recognition and management are essential to prevent further damage [6].

The percentage of the elevation of blood pressure over baseline and the severity of new-onset arterial hypertension are relevant due to the lack of adaptation of the blood-brain barrier to the hemodynamic changes of the hypertensive state. This adaptive mechanism is known as autoregulation of cerebral blood flow, consisting of the modification of cerebral vascular resistance (vasodilation/vasoconstriction) to ensure perfusion and meet metabolic demands. This autoregulation is determined by the partial pressure of carbon dioxide and mean arterial blood pressure and, to a lesser extent, by the partial pressure of oxygen, adenosine, pH, etc. [7]. When the threshold for cerebral perfusion pressure is exceeded, cerebral vasodilatation follows, leading to excessive perfusion with endothelial damage and edema. A compensatory mechanism consisting of autoregulatory vasoconstriction leads to hypoxia and cerebral ischemia, along with the subsequent increase in vascular endothelial growth factor and vasogenic edema, explaining the imaging findings of PRES [7].

Our patient had a normotensive generalized tonic-clonic seizure followed by a hypertensive crisis and neurological deterioration that resolved after pregnancy termination and blood pressure management. Since she had no previous history of arterial hypertension, it was hypothesized that her cerebral autoregulation mechanisms were overwhelmed by mild to moderate fluctuations or elevation above baseline blood pressure (explaining normotensive presentation) and later by high blood pressure levels (explaining subsequent cerebral and neurological deterioration) [8].

Hefzy et al. analyzed 151 patients and described some risk factors for PRES, including arterial hypertension, immunosuppression, sepsis, and preeclampsia [9]. Bartynski and Boardman evaluated the distribution of lesions in the images of 136 patients with PRES (22 had brain CT scan and 114 underwent cerebral MRI), showing a distribution of vasogenic edema (focal or confluent) on the white matter. The previous imaging patterns were defined as follows: (1) holo-hemispheric vasogenic edema (22.8%); (2) superior frontal sulcus vasogenic edema (27.2%); (3) parietal and occipital vasogenic edema (22.1%), a classic pattern of white matter involvement as reported on our patient’s MRI; and (4) partial or asymmetric vasogenic edema (27.9%) [10].

According to some publications, the location and distribution of edema may determine the severity. Based on fluid-attenuated inversion recovery (FLAIR) MRI, McKinney et al. defined mild cases as those in which the edema affects the cortical and subcortical white matter, moderate cases as those in which there is confluent edema extending from the cortex to the deep white matter, and severe cases as those in which there is confluent edema extending from the cortex to the ventricle, hemorrhage, midline shift, herniation, mass effect, involvement of the cerebellum, brain stem, or basal nuclei (DBRE) [11-16]. Therefore, this case could be classified as mild, which might explain our patient’s clinical improvement in response to treatment (Figure 1). As per consensus [5], labetalol or hydralazine are recommended as the first-line agents in the setting of severe hypertension, and other drugs such as nifedipine, nicardipine, and esmolol could also be administered. Our intervention consisted of utilizing labetalol, leading to an appropriate clinical response.

Hefzy et al. reported that despite its usual favorable outcome, this syndrome is associated with a mortality of 5-15% and that hemorrhagic transformation (petechiae, hematomas, subarachnoid hemorrhage, or intraventricular hemorrhage) increases mortality to 29%; based on this data, a high index of suspicion and timely diagnosis of PRES are essential for good clinical outcomes [9,17-19].

## Conclusions

We reported a case of a healthy 18-year-old G1P0 with an elevated risk for eclampsia presenting with PRES. Proteinuria and hypertension were absent on admission, but clinical syndrome and neuroimaging findings were indicative of a positive diagnosis. Autoregulation mechanisms of cerebral blood flow in patients with pregnancy-associated hypertensive disorders are very vulnerable since they can get overwhelmed due to a lack of adaptation. The relevance of the case report lies in the positive impact resulting from the timely identification of PRES associated with pregnancy even though it did not meet all the established criteria for HDP during its initial presentation. Early recognition and management of the condition can help prevent further damage.
